# Moiré excitons in MoSe_2_-WSe_2_ heterobilayers and heterotrilayers

**DOI:** 10.1038/s41467-021-21822-z

**Published:** 2021-03-12

**Authors:** Michael Förg, Anvar S. Baimuratov, Stanislav Yu. Kruchinin, Ilia A. Vovk, Johannes Scherzer, Jonathan Förste, Victor Funk, Kenji Watanabe, Takashi Taniguchi, Alexander Högele

**Affiliations:** 1 Fakultät für Physik, Munich Quantum Center, and Center for NanoScience (CeNS), Ludwig-Maximilians-Universität München, Geschwister-Scholl-Platz 1, 80539 München, Germany; 2 Center for Computational Materials Sciences, Faculty of Physics, University of Vienna, Sensengasse 8/12, 1090 Vienna, Austria; 3 Nuance Communications Austria GmbH, Technologiestraβe 8, 1120 Wien, Austria; 4 Center of Information Optical Technology, ITMO University, Saint Petersburg, 197101 Russia; 5 Research Center for Functional Materials, National Institute for Materials Science, 1-1 Namiki, Tsukuba, 305-0044 Japan; 6 International Center for Materials Nanoarchitectonics, National Institute for Materials Science, 1-1 Namiki, Tsukuba, 305-0044 Japan; 7 Munich Center for Quantum Science and Technology (MCQST), Schellingtraße 4, 80799 München, Germany

**Keywords:** Two-dimensional materials, Two-dimensional materials

## Abstract

Layered two-dimensional materials exhibit rich transport and optical phenomena in twisted or lattice-incommensurate heterostructures with spatial variations of interlayer hybridization arising from moiré interference effects. Here, we report experimental and theoretical studies of excitons in twisted heterobilayers and heterotrilayers of transition metal dichalcogenides. Using MoSe_2_-WSe_2_ stacks as representative realizations of twisted van der Waals bilayer and trilayer heterostructures, we observe contrasting optical signatures and interpret them in the theoretical framework of interlayer moiré excitons in different spin and valley configurations. We conclude that the photoluminescence of MoSe_2_-WSe_2_ heterobilayer is consistent with joint contributions from radiatively decaying valley-direct interlayer excitons and phonon-assisted emission from momentum-indirect reservoirs that reside in spatially distinct regions of moiré supercells, whereas the heterotrilayer emission is entirely due to momentum-dark interlayer excitons of hybrid-layer valleys. Our results highlight the profound role of interlayer hybridization for transition metal dichalcogenide heterostacks and other realizations of multi-layered semiconductor van der Waals heterostructures.

## Abstract (web summary)

Here, the authors show that the photoluminescence of MoSe_2_/WSe_2_ heterobilayers is dominated by valley-direct excitons, whereas, in heterotrilayers, interlayer hybridization turns momentum-indirect interlayer excitons into energetically lowest states with phonon-assisted emission.

## Introduction

Heterostructures of layered two-dimensional materials exhibit rich transport and optical phenomena. In twisted or lattice-incommensurate heterobilayers (HBLs), laterally modulated van der Waals interactions give rise to spatial variations in the degree of interlayer hybridization on the characteristic length scale of the moiré interference pattern^1–6^. The formation of moiré superlattices has profound effects on the electronic band structure, as evidenced by the emergence of correlated transport phenomena in flat bands of twisted bilayer^7,8^ and trilayer^9,10^ graphene, or detected optically in twisted homobilayers^11^ and aligned HBLs^12,13^ of transition metal dichalcogenides (TMDs). The latter also exhibit rich moiré signatures in the optical spectra of intralayer^14^ and interlayer^15–18^ excitons formed by Coulomb attraction among layer-locked and layer-separated electrons and holes.

In MoSe_2_-WSe_2_ HBL, a prominent representative of TMD heterostacks, the interlayer exciton photoluminescence (PL) is observed well below the intralayer features of monolayer MoSe_2_ and WSe_2_ constituents^19^. The PL energy is consistent with a staggered band alignment^20^ which separates electrons and holes into the conduction and valence bands of MoSe_2_ and WSe_2_, respectively. In accord with layer separation, interlayer excitons exhibit strongly prolonged radiative lifetimes^19^ and reduced oscillator strength^21^. Despite numerous experimental and theoretical studies of MoSe_2_-WSe_2_ HBLs, the origin of the lowest-energy PL remains a subject of debate^22^. While the majority of experimental studies interpret the HBL emission in terms of zero-momentum interlayer excitons with *K* or $K^{\prime}$ valley electrons and holes in MoSe_2_ and WSe_2_^15,16,19,21,23–29^, others invoke excitons built from hybridized HBL conduction band states at *Q* pockets^30–32^, located roughly halfway between the center of the first Brillouin zone at Γ and *K* or $K^{\prime}$ valleys. Band structure calculations indeed suggest that hybridization of states near *Q* conduction band and Γ valence band of MoSe_2_ and WSe_2_ gives rise to strong energy renormalization upon HBL formation^2,33,34^ which might turn either *QK* or *Q*Γ interlayer excitons, composed of electrons at *Q* and holes at *K* or Γ, into the lowest-energy states.

Additional complication arises in the presence of moiré effects. In moiré-modulated HBLs, electronic states exhibit valley-contrasting energy shifts upon interlayer hybridization, with states in *K* and $K^{\prime}$ valleys being less susceptible to energy-reducing interactions than the conduction band states at *Q* or the valence band states at Γ. This effect, analogous to the origin of the direct-to-indirect band gap cross-over in TMD monolayers and bilayers^35–37^, should also impact the band structure of HBLs^2^ yet has been mostly neglected in the context of moiré excitons^1,3–6^. Interlayer hybridization is expected to play an even more prominent role in heterotrilayer (HTL) systems with native homobilayers. For the explicit case of MoSe_2_-WSe_2_ HTLs, one would expect sizable hybridization effects between the MoSe_2_ bilayer band edge states at *Q* and their counterparts in monolayer WSe_2_, rendering the overall heterostructure an indirect band gap semiconductor.

## Results

### MoSe_2_-WSe_2_ heterobilayer and heterotrilayer in cryogenic spectroscopy

Motivated by the contrasting behavior anticipated for momentum-direct and indirect band edge interlayer excitons in MoSe_2_-WSe_2_ HBL and HTL, we performed optical spectroscopy studies of the corresponding moiré heterostructures on the same sample. The heterostack was connected to a charge-reservoir for voltage control of capacitive doping. To this end, a MoSe_2_ crystal with monolayer and bilayer regions was stacked onto a WSe_2_ monolayer by dry viscoelastic stamping^38^, encapsulated from both sides by hexagonal boron nitride (hBN) and stamped into contact with a gold electrode with gate voltage referenced against a grounded layer of silver capped by SiO_2_ (Supplementary Note 1). The MoSe_2_ crystal with a native bilayer region in 2H or AA′ stacking was twisted away from parallel R-type alignment by about 4^∘^ with respect to the WSe_2_ monolayer. At such relatively large angles, we expect the moiré heterostructure to be robust against reconstruction^39–41^ and thus to contrast previous studies of MoSe_2_-WSe_2_ HBLs carefully aligned for zero twist angle in R-type stacking^24,28^ as well as moiré-free HBLs obtained from chemical vapor deposition with lattice-mismatch relaxation and inherent alignment^21,23^.

**Fig. 1:**
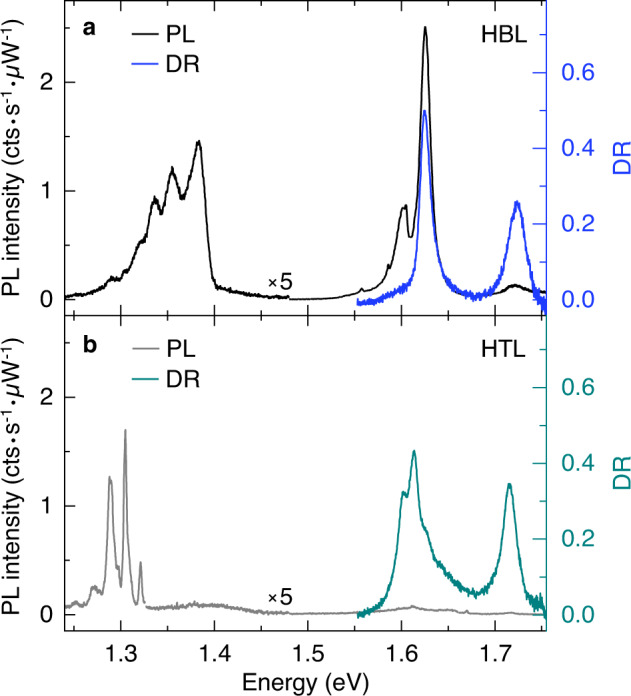
Cryogenic photoluminescence and differential reflectivity spectra of MoSe_2_-WSe_2_ heterobilayer and heterotrilayer. **a**, **b** Photoluminescence (black and gray) and differential reflectivity (blue and dark cyan) spectra of twisted HBL and HTL MoSe_2_-WSe_2_ at 3.2 K. The luminescence was excited with linearly polarized excitation laser at 1.85 eV and scaled in intensity below 1.47 eV by a factor of 5 in both graphs.

Cryogenic PL and differential reflectivity (DR) spectra of the HBL and HTL regions at 3.2 K and zero gate voltage are shown in Fig. 1a, b, respectively. The DR features in the spectral range between 1.55 and 1.75 eV are consistent with absorption characteristics of neutral intralayer excitons, and the vanishingly small trion feature in Fig. 1a indicates operation close to charge-neutrality (Supplementary Note 1). Whereas the two dominant DR peaks of the HBL spectrum in Fig. 1a essentially reflect the respective MoSe_2_ and WSe_2_ monolayer transitions around 1.6 and 1.7 eV, the HTL spectrum in Fig. 1b is different. Compared to the HBL spectrum, it exhibits a red-shift of the WSe_2_ intralayer exciton peak by 8 meV because of Coulomb screening by the additional MoSe_2_ layer, and a rich structure around the MoSe_2_ absorption peak with possible contributions from interlayer excitons of bilayer MoSe_2_^42^ as well as moiré miniband effects^6^ in the twisted HTL.

Within the same energy range, the cryogenic PL is consistently dominated by intralayer excitons. Remarkably, the intralayer MoSe_2_ and WSe_2_ peaks in the HBL spectrum of Fig. 1a are nearly completely quenched in the HTL spectrum of Fig. 1b, indicating for the latter drastically suppressed hot luminescence due to enhanced population relaxation into lowest-energy interlayer exciton levels. This observation is in accord with the theoretical prediction of increased charge transfer efficiency via hybridized *Q* and Γ states in heterostructures^2^.

Another striking difference in the PL of the heterostacks is evident in the spectra of Fig. 1a, b for interlayer excitons, with PL emission below 1.40 and 1.33 eV from HBL and HTL regions, respectively. The PL characteristics depend on the heterostack position, as confirmed by lateral displacement of the sample with respect to fixed confocal excitation and detection spots. Upon transition from the HBL to the HTL region, the set of the HTL peaks below 1.35 eV emerges at the expense of the higher-energy HBL peaks with emission energy above 1.35 eV (Supplementary Note 2). At each heterostack site, the overall multi-peak PL structure of HBL and HTL is mostly preserved upon the variation in the gate voltage (Supplementary Note 1) and excitation power down to 300 nW (Supplementary Note 3). Consistent with finite twist angle, the multi-peak PL of the HBL below 1.40 eV, with a peak separation of 30 meV between the two highest energy peaks and 15 meV between other consecutive peaks (Supplementary Note 3), is reminiscent of rich MoSe_2_-WSe_2_ moiré spectral features^16^ rather than of simple spectra from aligned HBLs^24–29^. Remarkably, the HTL PL, with a similar peak spacing of 15 meV, is strikingly similar to the cryogenic PL from native bilayer WSe_2_^43^ (Supplementary Note 4).

In time-resolved PL, HBL, and HTL PL exhibit similar decay dynamics (Supplementary Note 5). Spectral sampling of the PL decay characteristics indicates the presence of at least three decay channels without conclusive dependence on the emission energy for both HBL and HTL emission. Throughout the spectral band of interlayer excitons, the PL decay exhibits three decay timescales of 3, 12, and 480 ns for HBL and 1, 12, and 300 ns for HTL. For both heterostacks, PL decay was dominated by the slow component (with a weight of 89% and 80% in HBL and HTL, respectively) with contributions of the intermediate (fast) decay channel of 8% and 13% (3% and 7%) to the HBL and HTL emission, respectively. These timescales are in accord with previous studies of MoSe_2_-WSe_2_ HBL^21,31,44,45^ and subject to different and partly competing interpretations.

### Theory of excitons in R-stacked MoSe2-WSe2 heterobilayer and heterotrilayer

**Fig. 2:**
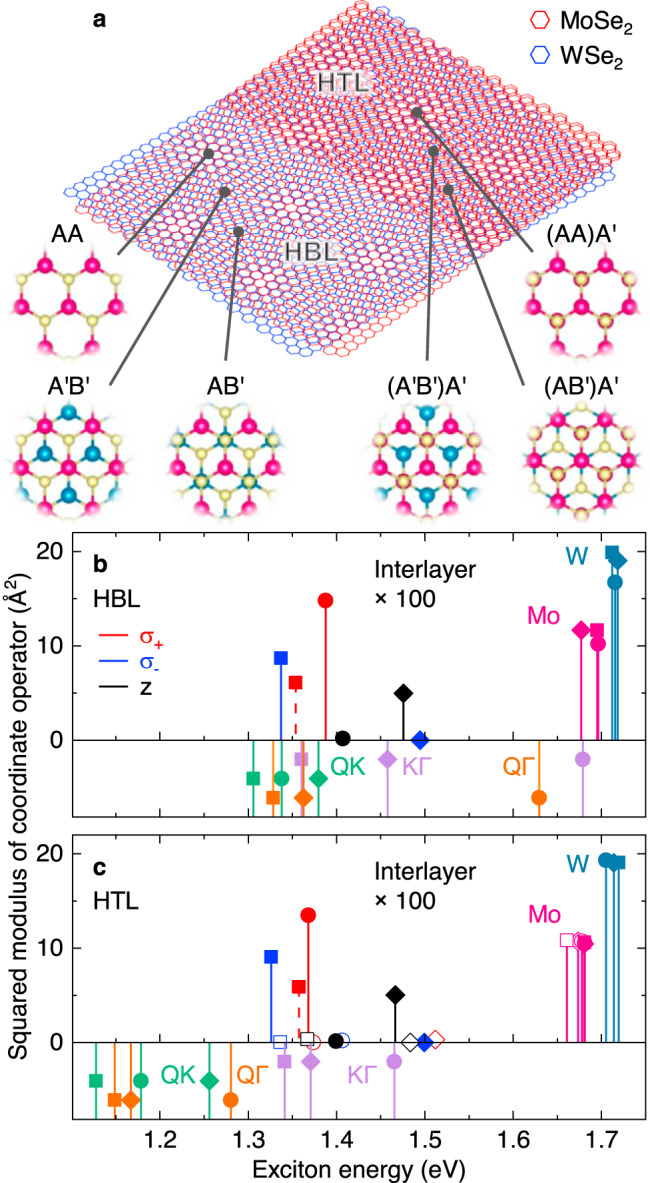
Theory of excitons in high-symmetry stackings of MoSe_2_-WSe_2_ heterobilayer and heterotrilayer. **a** Schematics of twisted HBL and HTL MoSe_2_-WSe_2_ with different high-symmetry stackings. **b**, **c** Energy and squared sum of coordinate operator (multiplied by 100 below 1.55 eV) for intralayer and interlayer excitons in HBL and HTL, respectively, calculated for three different stackings. Filled squares, circles, and diamonds denote A'B', AA, and AB', and (A'B')A', (AA)A', and (AB')A' stackings in HBL and HTL, respectively. Empty symbols indicate corresponding HTL excitons with electrons residing in the topmost MoSe_2_ layer. For zero-momentum *KK* interlayer excitons (top panels) we indicate the spin configuration by solid and dashed lines for spin-like and spin-unlike states (corresponding to spin-singlet and spin-triplet excitons), and the polarization of the respective exciton emission by red (*σ*^+^), blue (*σ*^−^), and black (in-plane *z*) colors. The bottom panels show the energy of finite-momentum interlayer excitons in *QK* (green), *Q*Γ (orange), and *K*Γ (violet) configurations without direct radiative transitions.

The differences in the PL spectra of Fig. 1a, b suggest different origins for the interlayer exciton PL in MoSe_2_-WSe_2_ HBL and HTL. To provide a basis for the interpretation of our observations, we performed numerical calculations of the band structure and exciton *g*-factors with density functional theory (DFT) in generalized gradient approximation (Supplementary Notes 6 and 7). Assuming that the twist angle is sufficiently small to employ local band structure approximation^4,46^, we restrict our analysis to three high-symmetry points of the moiré superlattice in each heterostructure with stackings indicated in Fig. 2a (Supplementary Note 6). Using the band structure results from DFT, we employed the Wannier exciton model in the effective mass approximation^47^ to calculate the energies of intralayer and interlayer excitons in different spin-valley configurations.

In the top panels of Fig. 2b, c we show the oscillator strength, essentially determined by the squared modulus of the coordinate operator matrix elements, for direct *KK* exciton transitions in different R-type stackings of HBL and HTL. For all stackings, interlayer excitons exhibit at least two orders of magnitude lower oscillator strengths than their intralayer counterparts^33^ with dipolar selection rules in agreement with the group theory analysis of R-type HBL^5,21^. In accord with previous calculations for HBLs, we find the lowest-energy *KK* interlayer exciton for A′B′^4,33^ and energetically higher excitons for AA and AB′ stackings. In all HBL stackings of R-type registry considered here, the lowest *KK* interlayer exciton is spin-like (i.e., in collinear electron spin orientation of occupied conduction band and unoccupied valence band states, and equivalent to spin-singlet configuration in the electron-hole notation), about 20 meV below its spin-unlike counterpart (or spin-triplet electron-hole pair). In AA stacking, the spin-like state has the largest oscillator strength, whereas for spin-unlike states only the *KK* exciton in A′B′ stacking has a sizable oscillator strength in agreement with previous DFT results^33,34^.

For the HTL, our calculations predict an increase in the number of conduction bands associated with lowest-energy excitons due to the additional MoSe_2_ layer. As such, interlayer *KK* excitons can be grouped according to the localization of the conduction band electron in one of the MoSe_2_ layers. For electrons localized on the MoSe_2_ layer with immediate proximity to WSe_2_ (full symbols in Fig. 2c), the corresponding interlayer excitons feature similar energies (with a small red-shift due to modified screening) and oscillator strengths as in the HBL system. Additional interlayer states arise from excitons with the electron localized in the upper MoSe_2_ layer (open symbols in Fig. 2c). Their energetic ordering, with spin-unlike configuration again being lowest, and dipolar selection are identical to *KK* interlayer excitons in HBLs of H-type registry^5,21^. However, the corresponding transitions have drastically inhibited oscillator strengths due to a reduced wavefunction overlap between the electron and hole in the topmost MoSe_2_ and the bottom WSe_2_ layer and thus should not contribute sizeably to the PL of HTL^48^. Based on our analysis, we rule out excitons composed of electrons and holes that are locked in distant layers as candidates for bright PL emission in the red-most part of the HTL spectrum.

In addition to *KK* excitons, our calculations yield the energies of momentum-indirect *QK*, *Q*Γ, and *K*Γ excitons (bottom panels of Fig. 2b, c) composed from electrons in *Q* (or $Q^{\prime}$) and *K* as well as holes at *K* or Γ. Note that the notion of oscillator strength is meaningless for momentum-indirect excitons without direct radiative decay pathways. The energetic ordering of interlayer excitons with zero and finite center-of-mass momentum differs substantially in HBL and HTL systems: whereas our calculations predict energetic proximity for *KK*, *QK,* and *K*Γ states in HBLs, finite-momentum *QK*, and *Q*Γ states in HTL are energetically well below the direct *KK* states, with an energy difference in the order of 200 meV. This trend is well known for monolayer and bilayer TMDs, where the states at *K* are much less sensitive to the addition of one layer than the states at *Q* and Γ^35–37,49^. For the HTL, strong interlayer hybridization should result in efficient layer coupling as opposed to layer locking^48^. The respective experimental signature of enhanced relaxation from intralayer to interlayer exciton states is the strong suppression of the HTL PL around the MoSe_2_ intralayer resonance at 1.62 eV in Fig. 1b.

### Power-dependent PL and degree of circular polarization of MoSe2-WSe2 heterobilayer and heterotrilayer

**Fig. 3:**
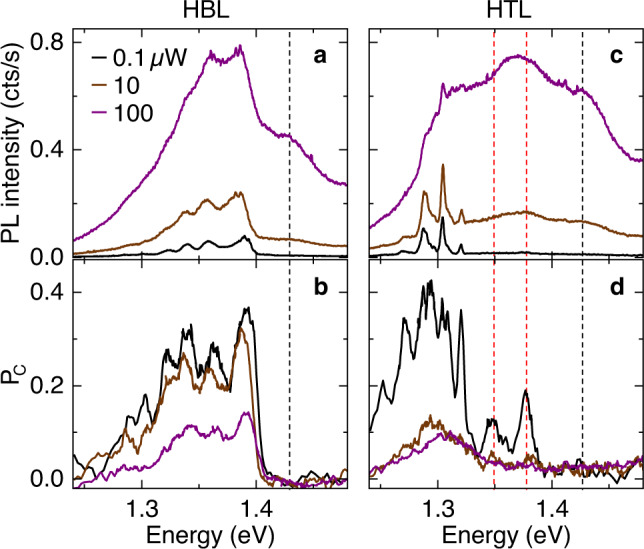
Power-dependent photoluminescence and degree of circular polarization of MoSe_2_-WSe_2_ heterobilayer and heterotrilayer. **a**, **b** Photoluminescence spectra and degrees of circular polarization (*P*_C_) for twisted HBL MoSe2-WSe2 at 0.1 (black), 10 (brown), and 100 *μ*W (purple) excitation power. **c**, **d** Same for HTL MoSe2-WSe2. Dashed lines indicate hot luminescence at the higher-energy side of the interlayer exciton spectrum with zero (black) and finite (red) *P*_C_.

We find experimental support for our theoretical description of HBL and HTL excitons by probing the PL and the degree of circular polarization (*P*_C_) as a function of excitation power. The corresponding results are shown in Fig. 3a–d for HBL and HTL, respectively. Upon increasing excitation power from 0.1 to 100 *μ*W, the HBL spectrum develops a pronounced shoulder above 1.40 eV with vanishing *P*_C_ (indicated by the dashed black line in Fig. 3a, b). This feature is consistent with hot luminescence from energetically higher states with *z*-polarized in-plane emission collected by our objective with high numerical aperture and corresponding collection solid angle. Our theory provides spin-unlike and spin-like interlayer excitons in AA and AB′ stacking, respectively, as two potential reservoirs for this emission (*z*-polarized states in Fig. 2b).

In contrast, the brightest HBL peaks between 1.32 and 1.40 eV with a positive degree of circular polarization are present down to lowest excitation powers. They are consistent with spin-like (spin-unlike) *KK* interlayer excitons in AA (A′B′) stacking (states in Fig. 2b with *σ*^+^ polarization). As we observe no sign reversal in *P*_C_ as expected for the lowest-energy spin-like *KK* interlayer exciton in A′B′ stacking (state in Fig. 2b with *σ*^−^ polarization) and reported previously for structured HBL PL^16^, strong contribution from A′B′ stacking is unlikely in our sample. This implies that AA domains dominate the HBL PL in rigid moiré supercells, although A′B′ regions should be at least of comparable size^39^. In the presence of reconstruction, one would expect predominance of energetically favored A′B′ and AB′ triangular domains of comparable area^39,40,50^. Without reconstruction, on the other hand, a reversal in the energetic ordering of AA and A′B′ interlayer excitons at finite twist angles, as predicted recently by theory for R-type MoSe_2_-WSe_2_ heterostructures^51^, would satisfactorily explain the observation.

According to our theory analysis, the structure of HTL PL is of different origin. The HTL data in Fig. 3c, d reveal *KK* interlayer exciton states through power-activated hot luminescence at the higher-energy side of the spectrum with finite circular degrees of polarization (indicated by the dashed red lines in Fig. 3c, d). The respective emission peaks at 1.35 and 1.38 eV can be ascribed to *KK* interlayer excitons with *σ*^+^ polarization in (A′B′)A′ or (AA)A′ stackings (red-colored states in Fig. 2c). Similar to the HBL, the HTL spectrum exhibits upon 100 *μ*W excitation power an additional higher-energy hot luminescence peak at 1.43 eV with vanishing *P*_C_ (black dashed line in Fig. 3c, d). The respective candidate from theory is the *KK* reservoir in (AB′)A′ stacking with *z*-polarized transition, as the (AA)A′ interlayer excitons with the same selection rules are dismissed due to vanishingly small oscillator strength (black-colored states in Fig. 2c). On the low energy side, the peaks around 1.30 eV below the energy of *KK* hot luminescence can result as phonon sidebands from momentum-dark *QK* or *Q*Γ interlayer exciton reservoirs (states in the bottom panel of Fig. 2c shown in green and orange). Without removing the ambiguity in the assignment of the lowest-energy reservoir to *QK* or *Q*Γ, this scenario explains the similarity in the spectral shape of HTL PL and the PL of native bilayer WSe_2_ originating from momentum-indirect excitons^43,52^.

### Magneto-luminescence of MoSe_2_-WSe_2_ heterobilayer and heterotrilayer

**Fig. 4:**
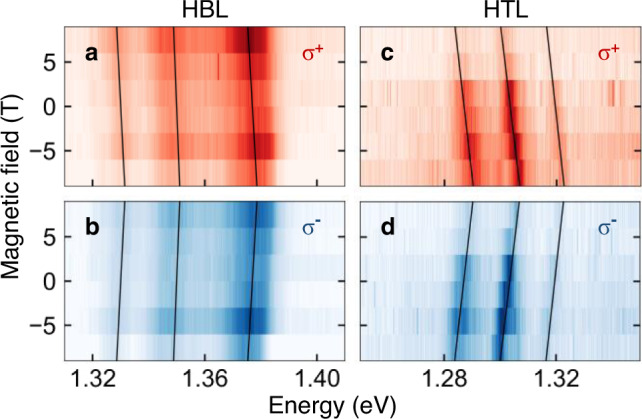
Valley Zeeman shift of interlayer excitons in MoSe_2_-WSe_2_ heterobilayer and heterotrilayer. **a**, **b** Magneto-luminescence of twisted HBL MoSe_2_-WSe_2_ for linear excitation and *σ*^+^ (red) and *σ*^−^ (blue) circularly polarized detection, respectively. **c**, **d** Same for HTL MoSe_2_-WSe_2_. The solid lines show magneto-induced energy shifts of HBL and HTL peaks with *g*-factors (from high to low energy) and error bars from least-square linear fits of − 6.2 ± 0.8, − 4.2 ± 0.8, and − 5.5 ± 0.8, and − 12.0 ± 2.0, − 12.0 ± 0.8, and − 13.0 ± 0.8, respectively.

Additional insight into the origin of HBL and HTL peaks is obtained from magneto-luminescence experiments in Faraday configuration and theoretical analysis. The experimental dispersion of the PL peaks in external magnetic field applied perpendicular to the heterostructure is shown in Fig. 4. The solid black lines indicate linear energy shifts recorded for *σ*^+^ and *σ*^−^ circularly polarized PL as a function of magnetic field. From this set of data, we determine the respective *g*-factors using the relation Δ*E* = *gμ*_*B*_*B*, where Δ*E* is the energy splitting between *σ*^+^ and *σ*^−^ polarized peaks proportional to the interlayer exciton *g*-factor, *μ*_*B*_ is the Bohr magneton, and *B* is the magnetic field. For the HBL, the extracted *g*-factors range between −4.2 and −6.2 with the same sign as for WSe_2_ intralayer excitons, whereas the HTL peaks exhibit *g*-factors between −12 and −13. In combination with observations described above and ab-initio calculations of *g*-factors for various spin-valley configurations of interlayer excitons in high-symmetry HBL and HTL stackings (Supplementary Note 7), these values suggest the following picture for MoSe_2_-WSe_2_ HBL and HTL stacks twisted away from ideal R-type registry.

## Discussion

First, we note that the experimental *g*-factors determined for the HBL peaks from the data of Fig. 4a, b (−6.2 ± 0.8, −4.2 ± 0.8, and −5.5 ± 0.8) are consistent with previous studies of aligned MoSe_2_-WSe_2_ heterostructures in R-type registry with absolute values in the range from 6.1 to 8.5^15,24,28^. They clearly contrast the interlayer exciton *g*-factor values between 15 and 16 in HBLs of H-type registry^15,26,29,53^. For the respective *KK* interlayer excitons in R-type HBLs, our calculations (Supplementary Note 7) predict an absolute *g*-factor value close to 6 and opposite signs for the degrees of circular polarization in AA and A′B′ stackings (with negative and positive *P*_C_, respectively), in agreement with a similar theoretical analysis of interlayer exciton *g*-factor values and signs^54,55^.

We proceed by discussing possible origins of the structured HBL emission. In the framework of moiré excitons, multi-peak PL has been ascribed to interlayer exciton states confined in moiré quantum wells^16,45^. Assuming that finite twist indeed reverses the energetic ordering of interlayer excitons in A′B′ and AA stackings^51^, the pronounced peaks of HBL PL below 1.40 eV with positive *P*_C_ and negative *g*-factors in the order of −6 would correspond to quantum well states of zero-momentum *KK* moiré excitons in AA stacked regions. This interpretation, however, is only plausible if the redistribution of interlayer exciton population among moiré quantum well sub-bands is bottlenecked. In the presence of population relaxation and thermal redistribution on timescales of the order or below 100–300 ns (longest decay components in PL), one would expect the PL from quantum well excited states to disappear or at least to diminish in intensity due to insufficient exciton population at low excitation powers and low temperature. Instead, we always find the highest energy peak to exhibit the highest intensity down to lowest excitation powers. An alternative interpretation invokes *KK* interlayer excitons trapped in disorder potentials. Within this scenario, the highest energy peak would stem from *KK* excitons in AA stacked regions decaying via their respective *σ*^+^ polarized radiative channel on the timescale of a few nanoseconds, whereas lower energy peaks with similar *g*-factors and *P*_C_ would reflect the respective defect-bound states with prolonged lifetimes. This scenario, however, is in conflict with the observation of spectrally independent PL decay, dismissing defect-trapped interlayer excitons with reduced energy as the primary source of the structured PL. The scenario of an energetically homogeneous distribution of localization by disorder over the entire spectral emission window seems even less plausible.

Finally, the multi-peak structure of HBL PL can be attributed to the joint emission from zero-momentum and finite-momentum interlayer exciton reservoirs^21,31^. In addition to spin-like *KK* excitons with a *g*-factor of 6, our theory identifies spin-like *K*Γ and spin-unlike $Q^{\prime} {{\Gamma }}$ with respective *g*-factors of 4 and 5 as candidates for the lower-energy HBL peaks. Note that theory finds these states in close energetic proximity to spin- and momentum-bright *KK* interlayer excitons (Fig. 2b). In this framework, the corresponding HBL peaks would qualify as phonon sidebands of *K*Γ or $Q^{\prime} {{\Gamma }}$ (or both), and the peak spacings of 30 and 15 meV would reflect the energies of optical and acoustic phonons, or higher-order combinations thereof^21,43^. This, however, holds only for staggered A′B′ and AB′ stackings that favor energy-reducing layer hybridization among the conduction band states around *Q* and $Q^{\prime}$ and the valence band states in the center of the Brillouin zone at Γ. In contrast, hybridization is less effective in AA stacked regions, upshifting the energies of *K*Γ and $Q^{\prime} {{\Gamma }}$ manifolds away from *KK* interlayer and towards intralayer excitons of MoSe_2_ and WSe_2_ (two highest energy states in the bottom panel of Fig. 2b). Remarkably, this setting predicts the contributions of zero-momentum and finite-momentum interlayer excitons to HBL PL to stem from different stackings and thus from spatially distinct reservoirs. Consequently, the two-dimensional landscape of lowest-energy moiré excitons would thus be shaped by momentum-direct and indirect states residing in spatially separated domains of different stackings.

The scenario is more simple for HTL PL with finite-momentum excitons being lowest in energy. For the peaks of Fig. 4c, d, the absolute values of *g*-factors of about 12 take momentum-direct *KK* interlayer excitons as well as momentum-indirect reservoirs *K*Γ and *Q*Γ out of the picture. Among the former, dipole-active *KK* states disqualify due to their *g*-factor of ~6, and *KK* spin-like (spin-unlike) configurations formed by the electron in the lower (upper) MoSe_2_ layer with theoretical *g*-factors between 11 and 13, as well as the respective $K^{\prime} K$ counterparts with similar *g*-factors, are dismissed due to higher energies and thus negligibly small exciton populations. The latter momentum-indirect *K*Γ and *Q*Γ states exhibit only small *g*-factors because of the vanishing valley Zeeman term in the Γ valley. By exclusion, the experimentally observed *g*-factors identify spin-like *QK* and spin-unlike $Q^{\prime} K$ interlayer excitons with theoretical *g*-factors of ~10 and 14 as the only reasonable sources for the HTL PL peaks in the form of phonon sidebands.

From the perspective of moiré exciton energy landscape governed by interlayer hybridization, lowest-energy HBL and HTL excitons should not differ in their spin-valley composition. Our analysis, however, suggests spin-like *K*Γ or spin-unlike $Q^{\prime} {{\Gamma }}$ states in HBL, and spin-like *QK* or spin-unlike $Q^{\prime} K$ states in HTL as lowest-energy manifolds. This controversy indicates that effects beyond hybridization have to be taken into account: the interplay of laterally modulated strain in moiré landscapes with opposite energy shifts for *K* versus *Q* and Γ valleys^56^, and the combined piezoelectric and ferroelectric effects in the order of tens of meV^51^ acting differently on interlayer excitons of distinct spin-valley configurations can reorder the hierarchy of energetically proximal interlayer exciton states.

In conclusion, our experimental and theoretical study of excitons in twisted MoSe_2_-WSe_2_ HBL and HTL of R-type registry promote a complex picture of HBL PL. It is consistent with radiative recombination of zero-momentum *KK* interlayer excitons and phonon-assisted emission from momentum-indirect reservoirs residing in spatially distinct regions of high-symmetry stackings. In contrast, the emission from the respective HTL system is entirely governed by phonon-assisted decay of momentum-dark *QK* or $Q^{\prime} K$ interlayer excitons. We base our conclusions on extensive optical spectroscopy experiments and calculations of the band structures, exciton states and *g*-factors for MoSe_2_-WSe_2_ HBL and HTL close to R-type registry. On these specific realizations of MoSe_2_-WSe_2_ heterostacks, our results highlight the primary role of moiré-modulated interlayer hybridization for the relaxation and formation of excitons in twisted van der Waals heterostructures with increasing layer number and structural complexity. Despite the extensive work presented here, a complete understanding of the rich phenomena observed in TMD heterostructures of different registries and alignment angles will require more efforts in experiment and theory to include piezoelectric and ferroelectric effects as well as strain at a detailed, quantitative level, and in the presence of reconstruction effects.

## Methods

The field-effect device based on a MoSe_2_-WSe_2_ heterostructure was fabricated by hot pick-up technique^38^. First, a layer of hBN was picked up, followed by MoSe_2_ with ML and BL regions, a ML of WSe_2_ and a capping layer of hBN. The heterostack was subsequently placed in contact to a gold electrode that was deposited on a silver-coated glass substrate with a SiO_2_ capping layer of 60 nm. PL and DR experiments were performed in a home-built cryogenic microscope. The sample was mounted on piezo-stepping and scanning units (attocube systems, ANPxy101, ANPz101, and ANSxy100) for positioning with respect to a low-temperature objective (attocube systems, LT-APO/LWD/NIR/0.63, or LT-APO/NIR/0.81). The microscope was placed in a dewar with an inert helium atmosphere at a pressure of 20 mbar and immersed in liquid helium at 4.2 K or operated at 3.2 K in a closed-cycle cryostat (attocube systems, attoDRY1000) equipped with a solenoid for magnetic fields of up to ±9 T. DR experiments were performed with a wavelength-tunable supercontinuum laser (NKT, SuperK Extreme, or SuperK Varia), also used for PL excitation around 633 or 715 nm with repetition rates down to 2 MHz. For continuous-wave measurements, the PL was excited with a laser diode at 635 nm or a HeNe laser, spectrally dispersed by a monochromator (Roper Scientific, Acton SP 2750, SP 2558, or Acton SpectraPro 300i) and recorded with a nitrogen-cooled silicon CCD (Roper Scientific, PyLoN, or Spec-10:100BR) or thermo-electrically cooled CCD (Andor iDus). Time-resolved PL was detected with an avalanche photodiode (Excelitas SPCM-AQRH) and correlated with a single photon counting system (PicoQuant, PicoHarp 300).

## Supplementary information

Supplementary Information

## Data Availability

The data that support the findings of this study are available from the corresponding authors upon reasonable request.
