# Supplementary material for: Moiré excitons in MoSe2-WSe2 heterobilayers and heterotrilayers
Source: Nat Commun. 2021 Mar 12;12:1656. doi: 10.1038/s41467-021-21822-z (PMC7955063; doi:10.1038/s41467-021-21822-z)
Supplement: Supplementary file 1 — Supplementary Information [file 41467_2021_21822_MOESM1_ESM.pdf]

SUPPLEMENTARY INFORMATION: Moiré excitons in $MoSe_2$-WSe_2 heterobilayers and heterotrilayers

Michael Förg,[1] Anvar S. Baimuratov,[1] Stanislav Yu. Kruchinin,[2,3] Ilia A. Vovk,[4] Johannes Scherzer,[1] Jonathan Förste,[1] Victor Funk,[1] Kenji Watanabe,[5] Takashi Taniguchi,[6] and Alexander Högele[1,7]

[1]*Fakultät für Physik, Munich Quantum Center, and Center for NanoScience (CeNS), Ludwig-Maximilians-Universität München, Geschwister-Scholl-Platz 1, 80539 München, Germany*

[2]*Center for Computational Materials Sciences, Faculty of Physics, University of Vienna, Sensengasse 8/12, 1090 Vienna, Austria*

[3]*Nuance Communications Austria GmbH, Technologiestraße 8, 1120 Wien*

[4]*Center of Information Optical Technology, ITMO University, Saint Petersburg 197101, Russia*

[5]*Research Center for Functional Materials, National Institute for Materials Science, 1-1 Namiki, Tsukuba 305-0044, Japan*

[6]*International Center for Materials Nanoarchitectonics, National Institute for Materials Science, 1-1 Namiki, Tsukuba 305-0044, Japan*

[7]*Munich Center for Quantum Science and Technology (MCQST), Schellingtraße 4, 80799 München, Germany*

Supplementary Note 1: Field effect characteristics of $MoSe_2$-WSe_2 heterostacks

The field effect $MoSe_2$-WSe_2 heterostructure shown in Supplementary Figure 1 was fabricated by standard mechanical exfoliation using the hot pick-up technique. First, a layer of high quality hexagonal boron nitride (hBN) was picked up with the stamp, followed by $MoSe_2$ with monolayer (ML) and bilayer (BL) regions, a ML of WSe_2 and a capping layer of hBN. The whole stack was subsequently deposited on a target substrate consisting of a silver (Ag) electrode with a protective layer of 60 nm SiO_2. The doping level in the entire $MoSe_2$-WSe_2 heterostructure was controlled by the gate voltage (V_g) applied to the Ag electrode in reference to a grounded gold electrode (Au) in contact with the WSe_2 ML.

The charge carrier doping was determined from voltage-dependent differential reflectivity (DR) of intralayer exciton transitions in HBL (Supplementary Figure 2a) and HTL (Supplementary Figure 3a). The corresponding voltage-dependent PL from interlayer excitons is shown in Supplementary Figure 2b and Supplementary Figure 3b, respectively. The domi-

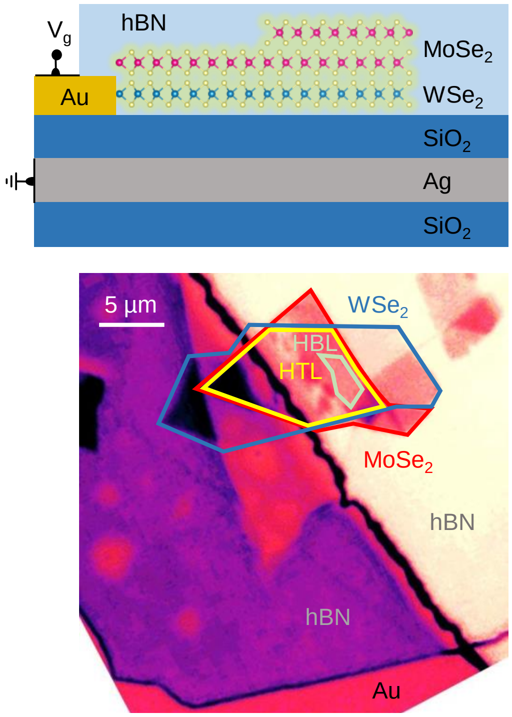

Supplementary Figure 1. Schematic layout (top) and optical micrograph (bottom) of the of the field effect heterostructure. $MoSe_2$ and WSe_2 few-layer crystals with monolayer and bilayer regions are delimited in red and blue, the resulting $MoSe_2$-WSe_2 HBL and HTL regions in light green and yellow, respectively.

nant $MoSe_2$ and WSe_2 ML features in the DR spectra of both Supplementary Figure 3a and Supplementary Figure 2a are related to the neutral intralayer excitons X_M and X_W, whereas the trion feature T_M in ML $MoSe_2$ is only weakly expressed. This observation characterizes the heterostacks as very close to the charge neutrality condition throughout the accessible voltage interval explored experimentally. Note that although the peak emission energy and intensity depend on the gate voltage, the overall spectral structure of the interlayer PL exhibits no significant changes. All data of the main text were recorded at 0 V.

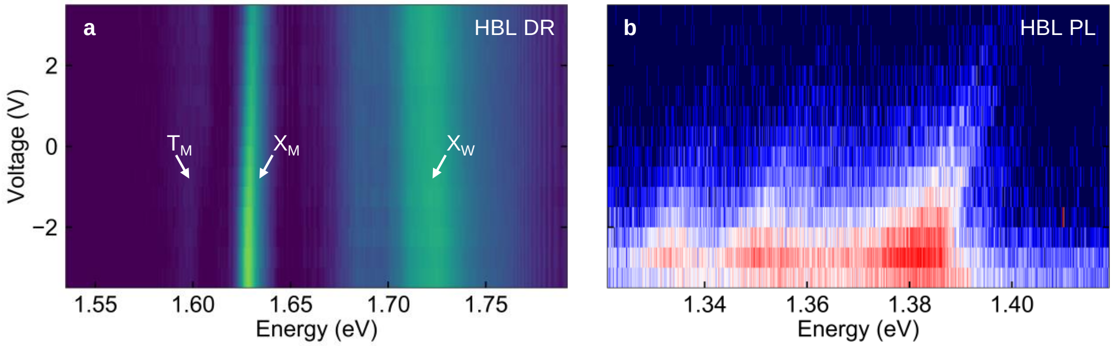

Supplementary Figure 2. **a**, Differential reflectivity (DR) of HBL in the spectral band of intralayer excitons as a function of gate voltage. The labelled features correspond to neutral excitons in ML $MoSe_2$ and WSe_2 (X_M and X_W, respectively) and a vanishingly weak ML $MoSe_2$ trion (T_M). **b**, Interlayer exciton PL recorded on the same HBL position. Note that the gate voltage does not change the overall spectral shape.

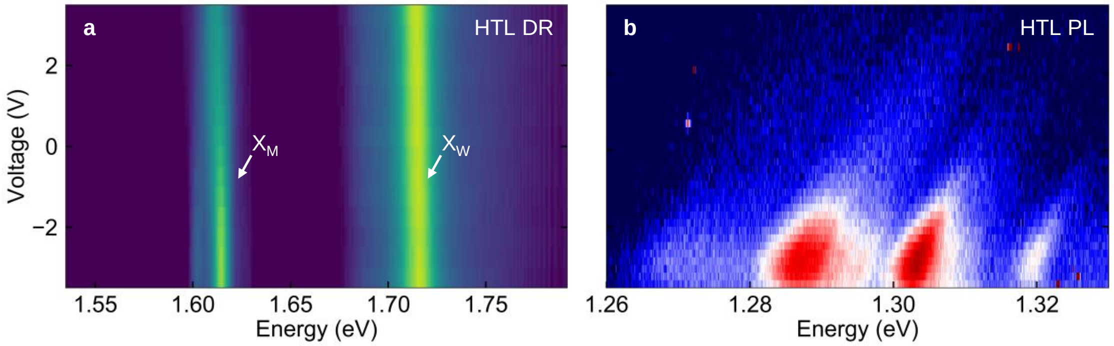

Supplementary Figure 3. **a**, Differential reflectivity (DR) of HTL in the spectral band of intralayer excitons as a function of gate voltage. The labelled features correspond to neutral excitons in ML $MoSe_2$ and WSe_2 (X_M and X_W, respectively). **b**, Interlayer exciton PL recorded on the same HTL position. Note that the gate voltage does not change the overall spectral shape.

Supplementary Note 2: Photoluminescence spectra as a function of lateral displacement from $MoSe_2$-WSe_2 heterobilayer to heterotrilayer

To confirm the reproducibility of the observations as a function of cooling cycles, the sample has been cool-cycled twice, using a low-temperature apochromatic objective with numerical aperture (NA) of 0.63 (with the respective focal spot diameter of ~ 1.5 μm) in the first run, and NA $= 0.81$ (with a smaller spot diameter of ~ 1.0 μm) in the second run. All data shown in the main text were recorded with the higher-NA objective. Supplementary Figure 4, with the data from the first run, exemplifies the dependence of the PL spectra on the lateral position. In this experiment, the sample was displaced with respect to fixed confocal excitation and detection spots by moving the heterostructure from the HBL to the HTL region. Evidently, the main features of HBL PL remain robust (peaks in the range $1.32 - 1.39$ eV in the bottom six spectra) before they entirely disappear on the HTL region where red-shifted peaks of HTL PL in the range $1.27 - 1.32$ eV (top four spectra) dominate. The weak yet finite PL cross-talk of HTL features into HBL spectra (that is further suppressed in Fig. 1 of the main text due to the smaller collection spot of the higher-NA objective) stems from insufficient lateral delimitation of the two regions with the objective of lower-NA.

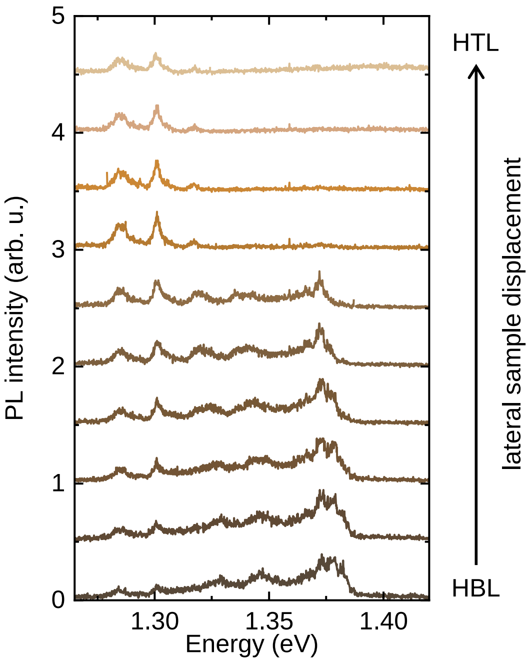

Supplementary Figure 4. PL spectra upon lateral sample displacement from the HBL (bottom) to the HTL region (top).

Supplementary Note 3: Dependence of photoluminescence multi-peak characteristics on gate voltage and excitation power

As discussed in the context of Supplementary Figure 3 and Supplementary Figure 2 the multi-peak PL structure is overall robust within the experimentally explored gate voltage range. The quantitative analysis of the spectra as a function of gate voltage recorded on a heterostructure region with contributions from both HBL and HTL stacks (as in the bottom spectrum of Supplementary Figure 4) is shown in Supplementary Figure 5. From the data in Supplementary Figure 5a it is evident that all peaks exhibit similar intensity variations, whereas the data in Supplementary Figure 5b and c confirm that the multi-peak PL structure is preserved throughout the gate voltage range between -1.5 and 1.5 V. In particular, the energy difference of 30 meV between the blue-most peaks (P1 and P2) and 15 meV between all other consecutive peaks is constant in the entire gate voltage range.

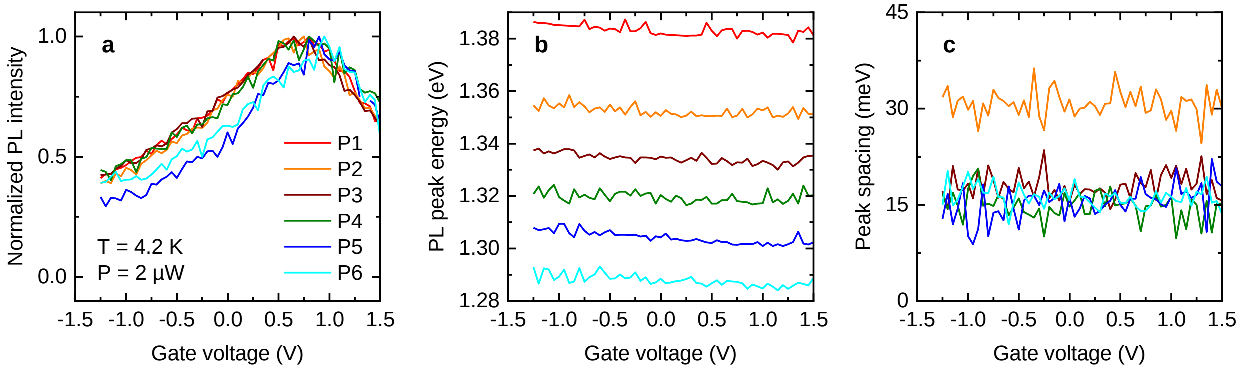

Supplementary Figure 5. **a** and **b**, Photoluminescence intensity and energy of peaks in the HBL and HTL spectra recorded on a sample position with contributions from both heterostacks. The peaks are numbered from P1 to P6 with decreasing emission energy. **c**, Energy difference between consecutive peaks as a function of gate voltage.

Qualitatively similar trends were observed as a function of excitation power in Supplementary Figure 6. The left and right panels of Supplementary Figure 6 show voltage-dependent HBL (the red-most peaks are due to cross-talk from the HTL region into the collection spot) and HTL PL for various laser excitation powers. Apart from power-dependent screening the set of data demonstrates that the characteristic multi-peak PL structures of both HBL and HTL are preserved over two orders of magnitude in excitation power. In particular, the intensity of the highest-energy HBL peak is highest at all powers, and the ratio to other peaks does not reverse down to the lowest excitation power of 0.3 μW.

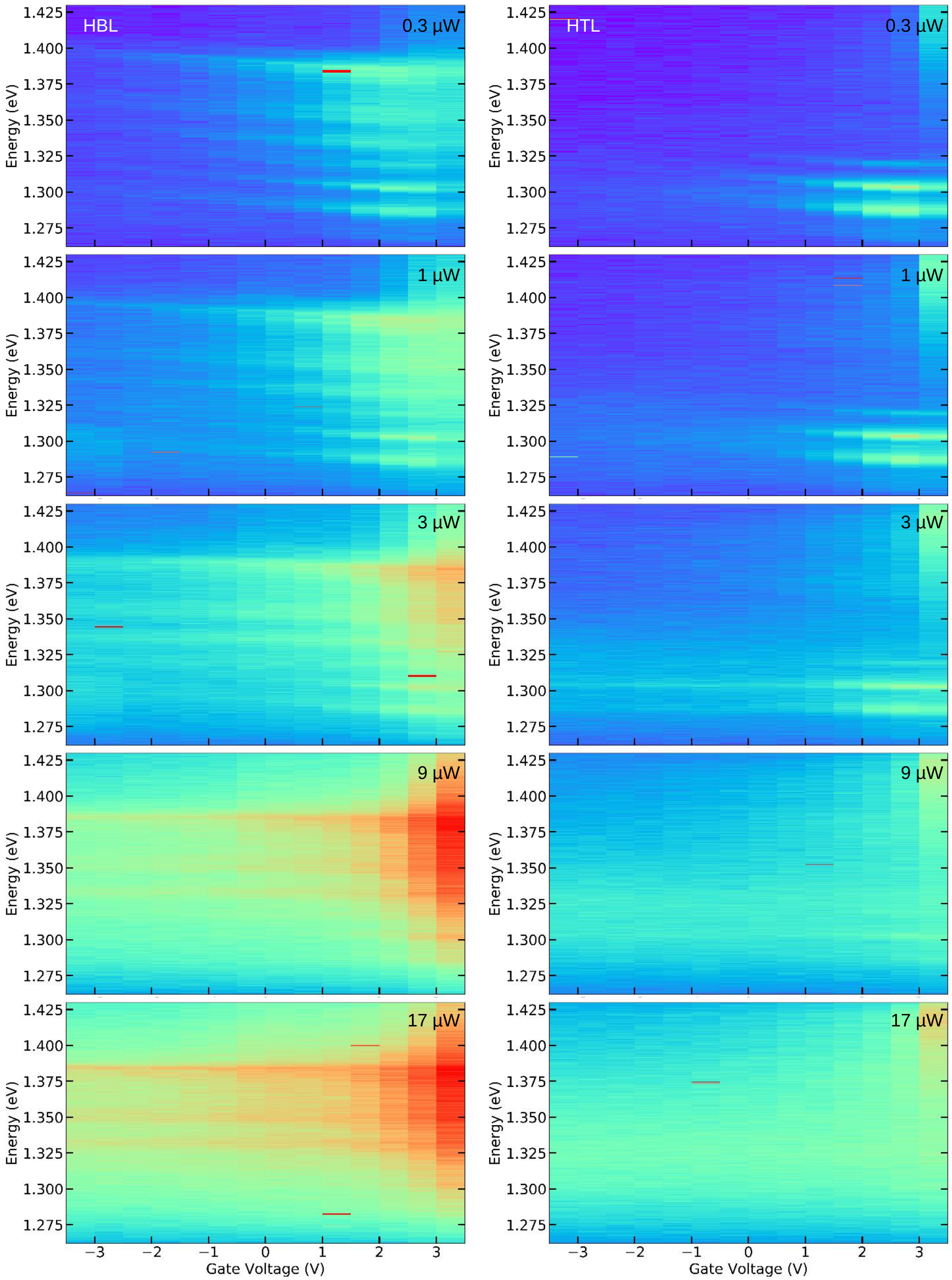

Supplementary Figure 6. HBL (left) and HTL (right) photoluminescence as a function of gate voltage at different excitation powers given explicitly in each graph.

Supplementary Note 4: Photoluminescence spectra of $MoSe_2$-WSe_2 heterotrilayer and native WSe_2 homobilayer

A striking similarity in the PL spectra from $MoSe_2$-WSe_2 HTL and native WSe_2 BL is evident from Supplementary Figure 9. Native WSe_2 BL exhibits PL emission as phonon-sidebands of momentum indirect QK excitons [1]. The spectra of $MoSe_2$-WSe_2 HTL and WSe_2 BL feature remarkably similar profiles when red-shifted by the band-offset energy of ~ 280 meV.

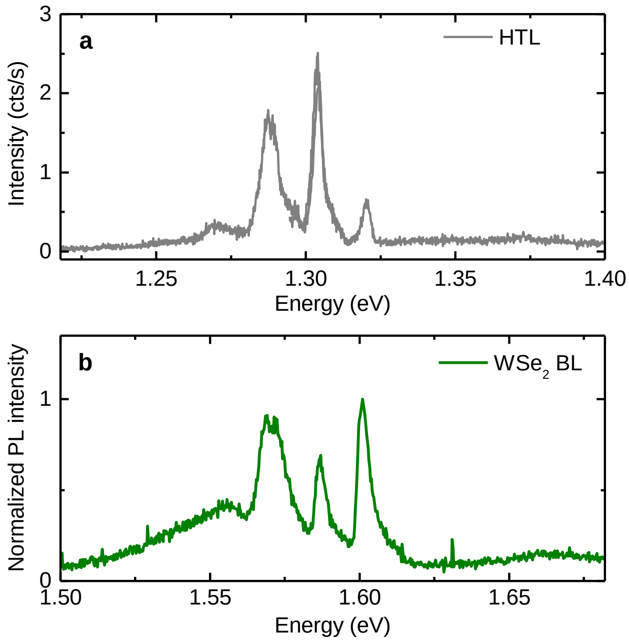

Supplementary Figure 7. **a**, Photoluminescence spectrum of interlayer excitons in $MoSe_2$-WSe_2 HTL and **b**, native WSe_2 BL encapsulated in hBN. For both measurements the temperature was 3.2 K.

Supplementary Note 5: Time-resolved photoluminescence decay

The PL dynamics of HBL and HTL PL were studied with time-resolved PL spectroscopy within various spectral bands. As evident from Supplementary Figure 8 and Supplementary Figure 9, the spectrally structured interlayer exciton emission of HBL and HTL exhibited similar PL decay characteristics. Good approximation to the PL decay was obtained with three exponential decay channels with lifetimes of 3, 12 and 480 ns for HBL and 1, 12 and 300 ns for HTL emission. Remarkably, the contributions of the individual decay channels to the total PL decay varied only marginally across the spectral bands. For both sample

regions, the decay was dominated by the slow decay component (with a weight of 89 and 80% in HBL and HTL, respectively) with contributions of the intermediate and fast decay channels of $\sim 10\%$ (with weights of 8 and 13% for the intermediate and 3 and 7% for the rapid components of HBL and HTL PL decay, respectively).

In Supplementary Figure 9, we summarize the decay data recorded in different spectral bands as indicated by colored regions in the top and bottom panels of Supplementary Figure 9a for HBL and HTL peaks, respectively. The decay times and weights of the three characteristic decay channels, obtained from tri-exponential decay fits within each spectral band, are summarized in Supplementary Figure 9b and c, respectively. The data suggest different PL decay characteristics within HBL and HTL regions with only little dependence on the emission energy for a given heterostack. This observation is inconsistent with lower PL peaks originating from exciton-localizing defects.

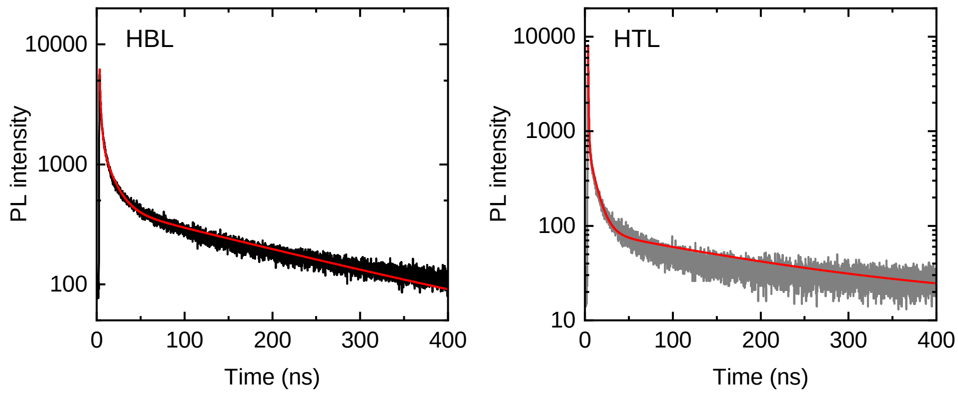

Supplementary Figure 8. Photoluminescence decay in $MoSe_2$-WSe_2 HBL (left) and HTL (right) at zero gate voltage. The excitation was performed at 633 nm and 1 μW power in the focal spot. The solid red lines show tri-exponential decay as best fits.

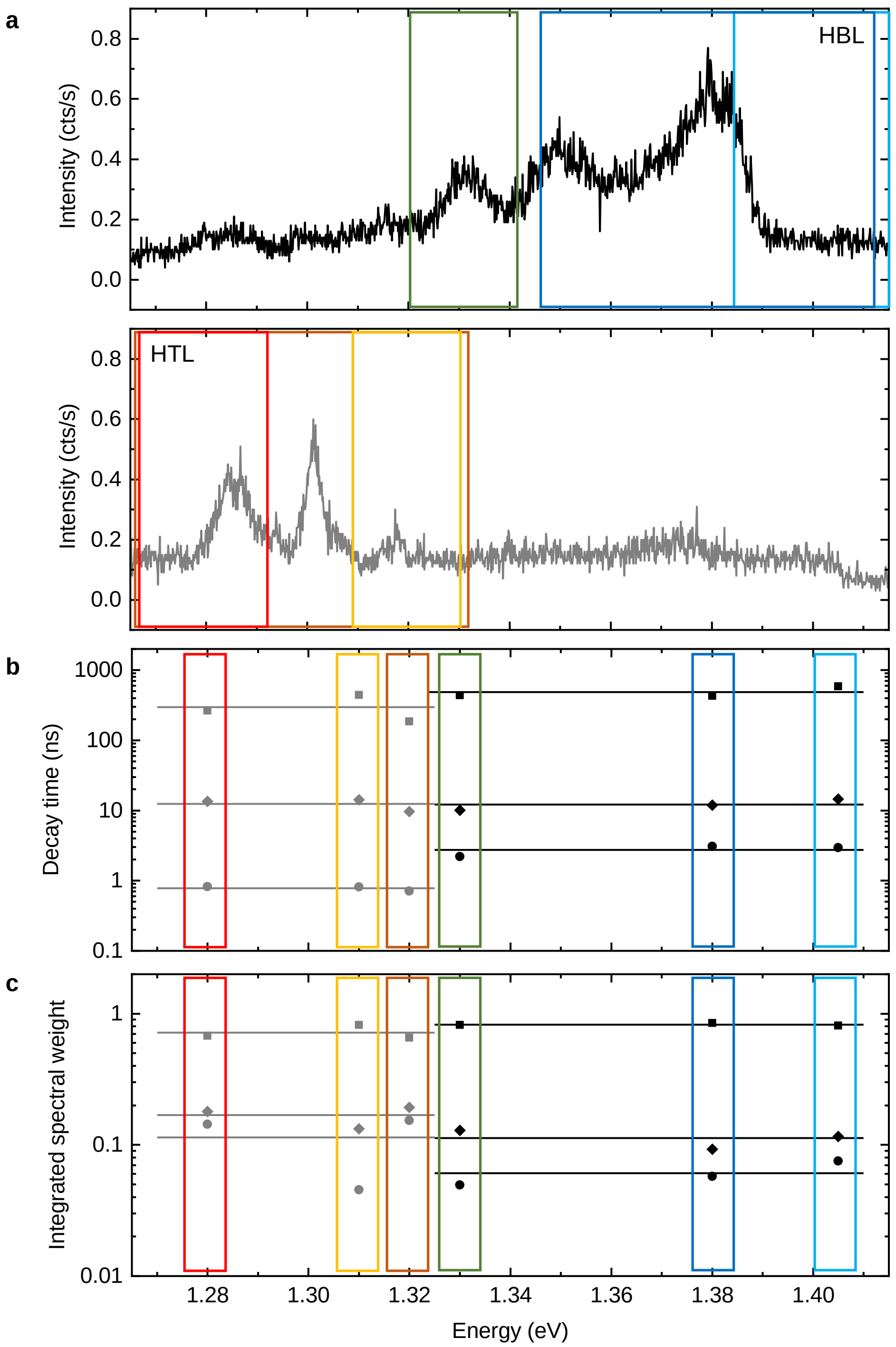

Supplementary Figure 9. **a**, Photoluminescence spectra of $MoSe_2$-WSe_2 HBL (top) and HTL (bottom) with colored spectral windows used to record time-resolved PL decay. **b** and **c**, Decay times and integrated spectral weights for three decay channels of the tri-exponential PL decay obtained from fits to spectrally limited PL bands of HBL (black data points, average values showed by black solid lines) and HTL (grey data points, average values showed by grey solid lines).

Supplementary Note 6: Exciton energies in $MoSe_2$-WSe_2 heterobilayers and heterotrilayers

Density functional theory (DFT) calculations of $MoSe_2$-WSe_2 HBL and HTL were performed with the PBEsol exchange-correlation functional [2] as implemented in the Vienna ab initio simulation package (VASP) [3]. Van der Waals interactions were included with the DFT-D3 method by Grimme *et al.* [4] with Becke-Johnson damping [5]. Moreover, spin-orbit interactions were included at all stages. Elementary cells with thickness of 35 Å in the z-direction were used in order to minimize interactions between periodic images. The atomic positions were relaxed with a cutoff energy of 400 eV until the total energy change was less than 10^{-6} eV. Calculations were performed for high-symmetry points of HBL and HTL moiré patterns in R-type stacking as shown in Supplementary Figure 10 on the Γ-centered $\mathbf{k}$ grid of 6×6 divisions with the cutoff energy of 300 eV, with 600 bands for the HBL and 900 bands for the HTL structures. The results for energy gaps and effective masses (in units of free electron mass m_0) are summarized in Supplementary Tables 1 and 2 for HBL and HTL.

Based on these DFT results, we used the Wannier exciton model in the effective mass approximation [6] to calculate the exciton energies E_X for different spin-valley configurations shown in Fig. 2 of the main text, obtained as $E_X = E_{\rm g}^{\rm DFT} + E_{\rm g}^{\rm offset} - E_{\rm b}$ from the DFT quasiparticle band gap energy $E_{\rm g}^{\rm DFT}$ corrected by an offset $E_{\rm g}^{\rm offset}$ that accounts for an underestimated band gap, and the exciton binding energy $E_{\rm b}$. $E_{\rm g}^{\rm offset} = 480$ meV was used as a global energy offset for all exciton configurations by placing experimental and theoretical energy positions of KK intralayer excitons of WSe_2 in resonance.

To determine the exciton binding energy we solved the stationary Schrödinger equation of the electron-hole relative motion,

$$\left[-\frac{\hbar^2}{2\mu}\nabla^2 + V(\rho)\right]\psi(\rho) = E_n\psi(\rho), \tag{1}$$

where $\psi(\rho)$ is the radial wave function, $\mu = m_{\rm e}m_{\rm h}/(m_{\rm e}+m_{\rm h})$ is the reduced effective mass, $m_{\rm e}$ and $m_{\rm h}$ are the effective masses of electron and hole, and $V(\rho)$ the Rytova–Keldysh potential [7, 8] of the form

$$V(\rho) = -\frac{\pi e^2}{2\varepsilon\rho_0}\left[H_0\left(\frac{\rho}{\rho_0}\right) - Y_0\left(\frac{\rho}{\rho_0}\right)\right], \tag{2}$$

with elementary charge e, effective dielectric constant ε, screening length ρ_0, and Struve and Neumann functions $H_0(x)$ and $Y_0(x)$. The binding energy was obtained as $E_{\rm b} = -\min(E_n)$

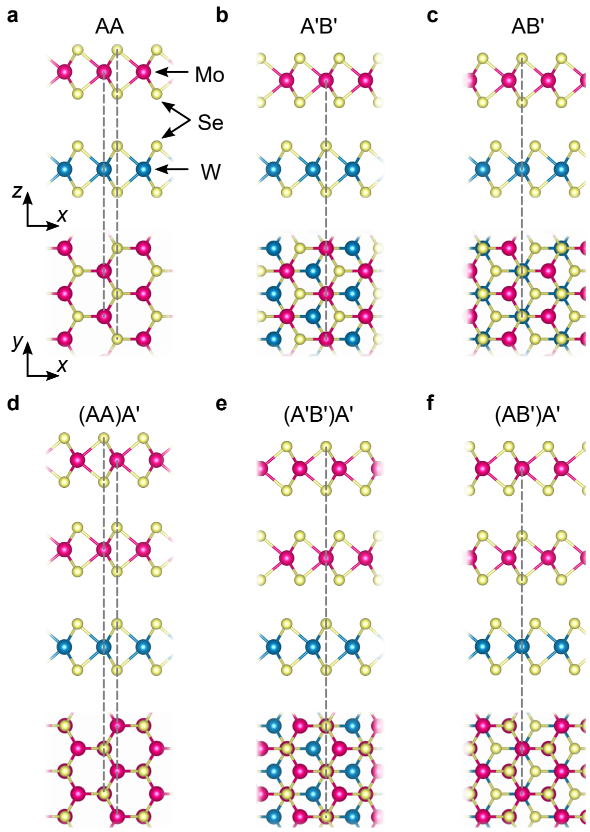

Supplementary Figure 10. High-symmetry stackings in R-type HBL (**a**–**c**) and HTL (**d**–**f**) $MoSe_2$-WSe_2.

from the minimal eigenvalue E_n, with $\varepsilon = 4.5$ and $\rho_0 = 1$ nm as parameters for $MoSe_2$ [9]. Remarkably, this procedure of calculating the exciton energy based on the Wannier model and DFT data yields good quantitative agreement with the computationally expensive simulations based on many-body approximations in HBL case [10]. However, different effective screening in the Coulomb interactions among electrons and holes in different valleys would result in relative energy shifts. A difference of ε by 10% for KK and QK states, for example, would yield an energy shift of about 35 meV. Presently, this uncertainty in the relative energy positions of interlayer exciton states in different valley configurations can not be eliminated from our theory as the valley-dependence of the effective dielectric screening is quantitatively unknown.

Supplementary Note 7: Calculation of exciton g-factors

Our methodology for calculations of exciton g-factors from first principles has been described in detail in [11]. Here, we recapitulate the main steps for determining the g-factors

Supplementary Table 1. Effective masses and energy gaps of HBL $MoSe_2$-WSe_2 from DFT.

Stacking	Electron k-point	Electron Layer	$m_e(m_0)$	Hole k-point	Hole Layer	$m_h(m_0)$	Energy gap E_g (eV)
AA	K	W	0.38	K	W	0.47	1.4
	K	Mo	0.67	K	Mo	0.74	1.43
	K	Mo	0.7	K	W	0.47	1.12
	K	Mo	0.67	K	W	0.47	1.1
	Q	Mo/W	0.66	K	W	0.47	1.05
	K	Mo	0.67	Γ	W/Mo	1.36	1.43
	Q	Mo/W	0.66	Γ	W/Mo	1.36	1.38
A'B'	K	W	0.37	K	W	0.44	1.39
	K	Mo	0.63	K	Mo	0.71	1.42
	K	Mo	0.71	K	W	0.44	1.06
	K	Mo	0.63	K	W	0.44	1.04
	Q	Mo/W	0.66	K	W	0.44	1.01
	K	Mo	0.63	Γ	W/Mo	0.85	1.09
	Q	Mo/W	0.66	Γ	W/Mo	0.85	1.06
AB'	K	W	0.39	K	W	0.46	1.4
	K	Mo	0.66	K	Mo	0.72	1.41
	K	Mo	0.73	K	W	0.46	1.2
	K	Mo	0.66	K	W	0.46	1.18
	Q	Mo/W	0.61	K	W	0.46	1.08
	K	Mo	0.66	Γ	W/Mo	0.92	1.20
	Q	Mo/W	0.61	Γ	W/Mo	0.92	1.10

of excitons in different spin and valley configurations in HBL and HTL $MoSe_2$-WSe_2. The exciton is formed by Coulomb correlations between an occupied state in the conduction band c with the wave vector $\mathbf{k}_c$ and spin z-projection s_c and an empty state in the valence band v with the wave vector $\mathbf{k}_v$ and spin z-projection s_v. In this spin-valley configuration, the exciton g-factor is given by

$$g^{(cv)}(\mathbf{k}_c, \mathbf{k}_v) = g_c(\mathbf{k}_c) - g_v(\mathbf{k}_v), \tag{3}$$

where the g-factor of the electron in band $n = c, v$ is

$$g_n(\mathbf{k}) = g_0 s_n + 2L_n(\mathbf{k}). \tag{4}$$

Here, $g_0 = 2$ is the free electron Landé factor, and the z-component of the orbital angular momentum [12–15] is

$$L_n(\mathbf{k}) = \frac{2m_0}{\hbar^2} \sum_{m \neq n} \mathrm{Im}\left[\xi^{(x)}_{nm}(\mathbf{k})\xi^{(y)}_{mn}(\mathbf{k})\right](E_{n\mathbf{k}} - E_{m\mathbf{k}}). \tag{5}$$

In the summation, the index m runs over all bands excluding the band of interest, $\boldsymbol{\xi}_{nm}(\mathbf{k}) = i\langle u_{n\mathbf{k}}|\partial/\partial\mathbf{k}|u_{m\mathbf{k}}\rangle$ is the interband matrix element of the coordinate operator, $E_{n\mathbf{k}}$ and $u_{n\mathbf{k}}$ are the energy and periodic Bloch amplitude of the electron in band n with wave vector $\mathbf{k}$.

Supplementary Table 2. Effective masses and energy gaps of HTL $MoSe_2$-WSe_2 from DFT.

	Electron			Hole			Energy gap
Stacking	k-point	Layer	$m_e(m_0)$	k-point	Layer	$m_h(m_0)$	E_g (eV)
(AA)A’	K	W	0.39	K	W	0.48	1.4
	K	Mo’	0.83	K	Mo’	0.79	1.42
	K	Mo	0.82	K	Mo	0.74	1.42
	K	Mo’	0.58	K	W	0.48	1.11
	K	Mo	0.6	K	W	0.48	1.1
	K	Mo’	0.83	K	W	0.48	1.09
	K	Mo	0.82	K	W	0.48	1.08
	Q	Mo/Mo’/W	0.57	K	W	0.48	0.88
	K	Mo	0.82	Γ	Mo/Mo’/W	0.80	1.21
	Q	Mo/Mo’/W	0.57	Γ	Mo/Mo’/W	0.80	1.01
(A’B’)A’	K	W	0.39	K	W	0.46	1.39
	K	Mo’	0.83	K	Mo’	0.75	1.42
	K	Mo	0.81	K	Mo	0.74	1.42
	K	Mo’	0.61	K	W	0.46	1.07
	K	Mo	0.58	K	W	0.46	1.06
	K	Mo’	0.83	K	W	0.46	1.05
	K	Mo	0.81	K	W	0.46	1.04
	Q	Mo/Mo’/W	0.53	K	W	0.46	0.83
	K	Mo	0.81	Γ	Mo/W/Mo’	0.94	1.09
	Q	Mo/Mo’/W	0.53	Γ	Mo/W/Mo’	0.94	0.88
(AB’)A’	K	W	0.38	K	W	0.44	1.4
	K	Mo’	0.81	K	Mo’	0.69	1.42
	K	Mo	0.79	K	Mo	0.73	1.4
	K	Mo’	0.63	K	W	0.44	1.21
	K	Mo	0.56	K	W	0.44	1.2
	K	Mo’	0.81	K	W	0.44	1.19
	K	Mo	0.79	K	W	0.44	1.18
	Q	Mo/Mo’/W	0.55	K	W	0.44	0.95
	K	Mo	0.79	Γ	Mo/W/Mo’	0.82	1.11
	Q	Mo/Mo’/W	0.55	Γ	Mo/W/Mo’	0.82	0.89

Using the energy band structure and interband matrix elements of the coordinate operator obtained from DFT we calculate the g-factor for excitons in different spin and valley configurations in HBL and HTL $MoSe_2$-WSe_2 according to the equations above. The results are summarized in Supplementary Table 3 for momentum-direct KK and momentum-indirect KK', QK, $Q'K$, $K\Gamma$, and $K'\Gamma$ interlayer excitons in HBL (top block) and HTL (bottom blocks) in different stackings of R-type registry.

Supplementary Table 3. Calculated g-factors of interlayer excitons in R-type $MoSe_2$-WSe_2 HBL (topmost block) and HTL (bottom blocks) in spin-like (↑↑) and spin-unlike (↓↑) configurations of conduction band electrons in K, K', Q or Q' valleys of $MoSe_2$ and empty valence band states at K in WSe_2 or at Γ in the hybrid band of $MoSe_2$-WSe_2. For each spin-valley configuration, the g-factors corresponding to the lower-energy state are shown in bold. For HTL, the upper (lower) block shows KK, $K'K$, $K\Gamma$, and $K'\Gamma$ excitons with the conduction band electron localized in the lower (upper) $MoSe_2$ layer, as well as QK, $Q'K$, $Q\Gamma$, and $Q'\Gamma$ excitons with small (large) hybridization with WSe_2 conduction band states. The sign convention for KK interlayer exciton g-factors is the same as for the KK intralayer exciton in WSe_2; only absolute values are given for momentum-indirect interlayer excitons as well as for direct KK excitons with z-polarized in-plane emission.

	KK		$K'K$		QK		$Q'K$		$K\Gamma$		$K'\Gamma$		$Q\Gamma$		$Q'\Gamma$	
Stacking	↑↑	↓↑	↑↑	↓↑	↑↑	↓↑	↑↑	↓↑	↑↑	↓↑	↑↑	↓↑	↑↑	↓↑	↑↑	↓↑
AA	**−6.4**	11.0	13.0	**17.6**	**9.0**	13.3	10.7	**15.0**	**3.6**	1.0	3.0	**7.6**	**1.0**	3.3	0.7	**5.0**
A'B'	**+5.8**	−10.5	13.1	**17.8**	**8.6**	13.0	10.6	**14.9**	**4.0**	0.7	3.3	**8.0**	**1.1**	3.2	0.8	**5.1**
AB'	**6.3**	+10.9	13.0	**17.6**	**8.7**	12.9	11.0	**15.3**	**3.7**	1.0	3.0	**7.7**	**1.3**	2.9	1.1	**5.3**
(AA)A'	**−6.3**	11.6	12.6	**17.9**	**9.9**	14.1	10.1	**14.3**	**3.8**	1.5	2.5	**7.8**	**0.2**	4.0	0.0	**4.2**
(A'B')A'	**+5.9**	−10.8	13.1	**17.9**	**9.5**	13.8	10.1	**14.4**	**4.0**	0.8	3.2	**8.0**	**0.4**	3.8	0.2	**4.4**
(AB')A'	**6.3**	+12.2	12.0	**18.0**	**9.6**	13.9	10.4	**14.6**	**3.8**	2.1	1.9	**7.8**	**0.5**	3.7	0.3	**4.5**
(AA)A'	+12.8	**−18.1**	**6.1**	11.4	9.1	13.3	10.9	15.1	2.7	**8.0**	**4.0**	1.3	1.0	3.2	0.8	5.0
(A'B')A'	13.1	**+18.0**	**5.9**	10.7	8.8	12.9	10.9	15.1	3.2	**8.0**	**4.0**	0.8	1.2	3.0	1.0	5.2
(AB')A'	−12.9	**18.8**	**5.5**	11.4	9.1	12.9	11.3	15.1	2.8	**8.7**	**4.7**	1.2	1.0	2.8	1.2	5.0

SUPPLEMENTARY REFERENCES

[1] J. Lindlau, M. Selig, A. Neumann, L. Colombier, J. Förste, V. Funk, M. Förg, J. Kim, G. Berghäuser, T. Taniguchi, K. Watanabe, F. Wang, E. Malic, and A. Högele, The role of momentum-dark excitons in the elementary optical response of bilayer WSe_2, Nat. Commun. **9**, 2586 (2018).

[2] G. I. Csonka, J. P. Perdew, A. Ruzsinszky, P. H. T. Philipsen, S. Lebègue, J. Paier, O. A. Vydrov, and J. G. Ángyán, Assessing the performance of recent density functionals for bulk solids, Phys. Rev. B **79**, 155107 (2009).

[3] M. Shishkin and G. Kresse, Self-consistent GW calculations for semiconductors and insulators, Phys. Rev. B **75**, 235102 (2007).

[4] S. Grimme, J. Antony, S. Ehrlich, and H. Krieg, A consistent and accurate ab initio parametrization of density functional dispersion correction (DFT-D) for the 94 elements H-Pu, J. Chem. Phys. **132**, 154104 (2010).

[5] S. Grimme, S. Ehrlich, and L. Goerigk, Effect of the damping function in dispersion corrected density functional theory, J. Comp. Chem. **32**, 1456 (2011).

[6] G. Berghäuser and E. Malic, Analytical approach to excitonic properties of MoS_2, Phys. Rev. B **89**, 125309 (2014).

[7] L. V. Keldysh, Coulomb interaction in thin semiconductor and semimetal films, JEPT Lett. **29**, 658 (1979).

[8] N. S. Rytova, The screened potential of a point charge in a thin film, Mosc. Univ. Phys. Bull. **3**, 18 (1967).

[9] B. Han, C. Robert, E. Courtade, M. Manca, S. Shree, T. Amand, P. Renucci, T. Taniguchi, K. Watanabe, X. Marie, L. E. Golub, M. M. Glazov, and B. Urbaszek, Exciton states in monolayer $MoSe_2$ and $MoTe_2$ probed by upconversion spectroscopy, Phys. Rev. X **8**, 031073 (2018).

[10] R. Gillen and J. Maultzsch, Interlayer excitons in $MoSe_2/WSe_2$ heterostructures from first principles, Phys. Rev. B **97**, 165306 (2018).

[11] J. Förste, N. V. Tepliakov, S. Yu. Kruchinin, J. Lindlau, V. Funk, M. Förg, K. Watanabe, T. Taniguchi, A. S. Baimuratov, and A. Högele, Exciton g-factors in monolayer and bilayer WSe_2 from experiment and theory, Nat. Commun. **11**, 4539 (2020).

[12] L. M. Roth, B. Lax, and S. Zwerdling, Theory of optical magneto-absorption effects in semiconductors, Phys. Rev. **114**, 90 (1959).

[13] G. L. Bir and G. E. Pikus, *Symmetry and strain-induced effects in semiconductors* (Wiley New York, 1974).

[14] D. Xiao, M.-C. Chang, and Q. Niu, Berry phase effects on electronic properties, Rev. Mod. Phys. **82**, 1959 (2010).

[15] G. Wang, L. Bouet, M. M. Glazov, T. Amand, E. L. Ivchenko, E. Palleau, X. Marie, and B. Urbaszek, Magneto-optics in transition metal diselenide monolayers, 2D Mater. **2**, 034002 (2015).
